# *CCL3L1* copy number and susceptibility to malaria

**DOI:** 10.1016/j.meegid.2012.03.021

**Published:** 2012-07

**Authors:** Danielle Carpenter, Anna Färnert, Ingegerd Rooth, John A.L. Armour, Marie-Anne Shaw

**Affiliations:** a Centre for Genetics and Genomics, School of Biology, University of Nottingham, Nottingham NG7 2UH, UK; b Infectious Disease Unit, Department of Medicine Solna, Karolinska Institutet, Stockholm, Sweden; c Nyamisati Malaria Research, Rufiji, National Institute for Medical Research, Dar-es-Salaam, Tanzania; d Institute of Integrative and Comparative Biology, Faculty of Biological Sciences, University of Leeds, Leeds LS2 9JT, UK

**Keywords:** *CCL3L1*, MIP-1α, Malaria, Haplotype

## Abstract

Copy number variation can contribute to the variation observed in susceptibility to complex diseases. Here we present the first study to investigate copy number variation of the chemokine gene *CCL3L1* with susceptibility to malaria. We present a family-based genetic analysis of a Tanzanian population (*n* = 922), using parasite load, mean number of clinical infections of malaria and haemoglobin levels as phenotypes. Copy number of *CCL3L1* was measured using the paralogue ratio test (PRT) and the dataset exhibited copy numbers ranging between 1 and 10 copies per diploid genome (pdg). Association between copy number and phenotypes was assessed. Furthermore, we were able to identify copy number haplotypes in some families, using microsatellites within the copy variable region, for transmission disequilibrium testing. We identified a high level of copy number haplotype diversity and find some evidence for an association of low *CCL3L1* copy number with protection from anaemia.

## Highlights

► We model, for the first time, *CCL3L1* copy number variation and susceptibility to malaria. ► Association analysis was performed using family-based methods in a Tanzanian population. ► We question whether malaria has shaped the current spectrum of variation observed at *CCL3L1.* ► We identify a weak association between *CCL3L1* copy number and haemoglobin concentration.

## 1 Introduction

Malaria caused by *Plasmodium falciparum* is a major cause of mortality and morbidity, especially in sub-Saharan Africa. Individuals who live in endemic areas may be subjected to constant exposure to infective mosquito bites and depending on the level of immunity, develop parasitaemia (parasites in the blood) and/or clinical disease. Variations in disease pattern can be attributable to a number of factors, including host genetic background, which was recently shown to account for approximately a quarter of the total variation observed in susceptibility to malarial diseases (Mackinnon et al., 2005).

Resolution of *P. falciparum* infection requires the production of cytokines, and the activation of macrophages leading to the destruction of parasites (Luty et al., 2001). Activation of macrophages and the recruitment to sites of infection is through chemoattractant cytokines, including macrophage inflammatory protein (MIP-1α, a small, low molecular weight, β-chemokine. This chemokine is secreted from most mature leucocytes and acts as a pro-inflammatory cytokine, being inhibited by IL-4, IL-10 and IL-13 (Berkman et al., 1995; Standiford et al., 1993).

The genes *CCL3* and *CCL3L1* encode the MIP-1α isoforms LD78α and LD78β respectively. The mature MIP-1α isoforms differ at only 3 amino acids, at positions 3, 39 and 47, with the isoform LD78β being 2-fold more efficient at chemoattracting human monocytes and lymphocytes than the LD78α isoform (Menten et al., 1999). The gene *CCL3L1* is copy variable and is located on chromosome 17q12 in a repeat unit of approximately 90 kb. Each repeat unit contains a single copy of *CCL3L1* and a single copy of another copy variable chemokine gene, *CCL4L1*, and is flanked by the gene *TBC1D3*. The repeat unit neighbours the paralogous, but copy invariant, genes *CCL3* and *CCL4* (Irving et al., 1990), and is thought to have evolved by duplication of the *CCL3* and *CCL4* region and subsequent divergence. Consequently *CCL3* and *CCL3L1* and also *CCL4* and *CCL4L1* exhibit a high degree (96%) of both nucleotide and protein sequence identity.

The *CCL3L1*/*CCL4L1* copy-variable region is of further interest as it varies markedly between populations; European populations exhibit a range of between 0 and 5 copies per diploid genome (pdg), with a modal copy of 2 (Carpenter et al., 2011; Gonzalez et al., 2005; Townson et al., 2002), whereas African populations have a greater range of between 2 and 10 pdg, with a modial copy of 4 (Gonzalez et al., 2005). It is not known why there is such divergence in copy number between populations but it has been suggested that there could be some recent population-specific selective force acting (Redon et al., 2006).

MIP-1α has previously been shown to play a role in protozoan infections; an increase in MIP-1α expression is observed in response to *Toxoplasma gondii* (Bliss et al., 1999) and *Leishmania* (Ritter et al., 1996; Teixeira et al., 2005). Serum concentrations of MIP-1α were shown to increase to a maximum 14 days post diagnosis in Thai malaria patients, suggesting that whilst MIP-1α is not involved in the acute phase of malaria infection, it may play a role in the later phase and the development of immunity (Burgmann et al., 1995). Furthermore MIP-1α protein and mRNA expression have been shown to be significantly elevated in the plasma of children with both mild and severe malaria (Ochiel et al., 2005), inducing proliferation of macrophages and expression of TNF-α, and exposure to *P. falciparum* antigens enhances MIP-1α production in neonates (Kocherscheidt et al., 2010). There is also evidence that MIP-1α inhibits haematopoietic stem cell proliferation (Graham and Pragnell, 1992; Graham et al., 1990), which may contribute to the malarial anaemia phenotype.

The involvement of MIP-1α in the pathology of *P. falciparum* infection makes malaria an attractive candidate to have exerted significant selective pressure on *CCL3L1* copy number in the past. Here we investigate whether copy number variation of *CCL3L1* may play a role in susceptibility to parasite prevalence and high loads, mean number of clinical episodes of malaria and haemoglobin levels in a longitudinal study of a rural Tanzanian population living in the Rufiji river delta. Furthermore, as the data set allows family based analysis this will allow us to use segregation to dissect the different haplotype backgrounds to allow transmission disequilibrium testing (TDT) analysis.

This method of genotyping *CCL3L1* copy number has previously only been used on European samples where it has been shown to be an accurate and reproducible method to genotype copy number (Carpenter et al., 2011; Walker et al., 2009). However, African samples exhibit higher copy number values (Gonzalez et al., 2005) and potentially a more complex pattern of variation at this locus (Walker et al., 2009). As such the PRT method was further developed for use with African samples.

## 2 Materials and methods

### 2.1 Study population

The study population is the well described Tanzanian Bantu population from the fishing village of Nyamisati, in the Rufiji river delta, 150 km south of Dar-es-Salaam (Carpenter et al., 2007, 2009; Rooth, 1992). The region was holoendemic for malaria with transmission increasing during the two rainy seasons (April–June and November–December). The predominant malaria species is *P. falciparum* and parasite prevalence in 1993 was 75% falling to 48% in 1998 as measured by microscopy in the 2–9 year old children. Cerebral malaria was not observed in the village during 1985–1999 when a research team provided health care in the village.

Human genomic DNA was extracted from frozen packed cells sampled in EDTA from a total of 1050 samples, as previously described (Carpenter et al., 2007, 2009) with informed consent and approval of the local ethics committees by the National Institute of Medical Research of Tanzania and the Regional Ethical Committee of Stockholm, Sweden. There were 15 individuals in the village known to be positive for HIV-1; for health and safety reasons DNA was not extracted from these samples, and these samples are excluded from any genetic analysis.

### 2.2 Phenotypes

The three clinical phenotypes (parasite load, mean number of clinical episodes of malaria and haemoglobin levels) were generated from data collected in Nyamisati from 1993 to 1999, using annual non-selected ‘total’ population surveys carried out during March and April, and a complete annual record of clinical malaria episodes all documented and collected by I.R., living in the village since 1985 (Rooth, 1992). The parasite load and mean number of clinical episodes of malaria data have previously been described in detail (Carpenter et al., 2009); in brief, the annual population surveys provided longitudinal data on ‘asymptomatic’ parasite densities (parasites per μl blood) recorded between March and April. The annual records gave the total number of clinical episodes each year per year per person, defined as fever (>37.5 °C) together with >5000 *P. falciparum* parasites/μl blood on microscopy. To generate a quantitative trait the data for both these phenotypes was log-transformed and corrected for age and sex for each year that data was collected (1993–1999). An arithmetic mean of the residuals generated from this correction for each year (1993–1999) was calculated to produce a single quantitative value for asymptomatic parasite load (p/μl) and for mean number of clinical episodes of malaria for use in the genetic analysis (Carpenter et al., 2009).

The population surveys contain information on haemoglobin concentration (g/l) for years 1994 to 1999. The majority of the 1289 individuals recorded in the population surveys do not have data from all years, but individuals have an average of 1.75 entries (range 1–4). To generate a single quantitative trait from the longitudinal haemoglobin data the mean haemoglobin was calculated per sample. Age and sex correction was carried out annually using a general linear model with age, age^2^ and sex as covariates. A small proportion (1%) of these individuals also presented with clinical episodes at the time of survey, when cross referenced with the clinical episode records for March to May, and for these samples only the peak parasite load (parasite/μl) was also included as a covariate in the general linear model to take into account the effect of parasite load on haemoglobin values. The residuals were recorded for each year (1994–1999) and the arithmetic mean was subsequently calculated to give a single quantitative value for haemoglobin level (see Fig. 3) (Carpenter, 2003).

### 2.3 Copy number measurement

**Supplementary Fig. 1:**
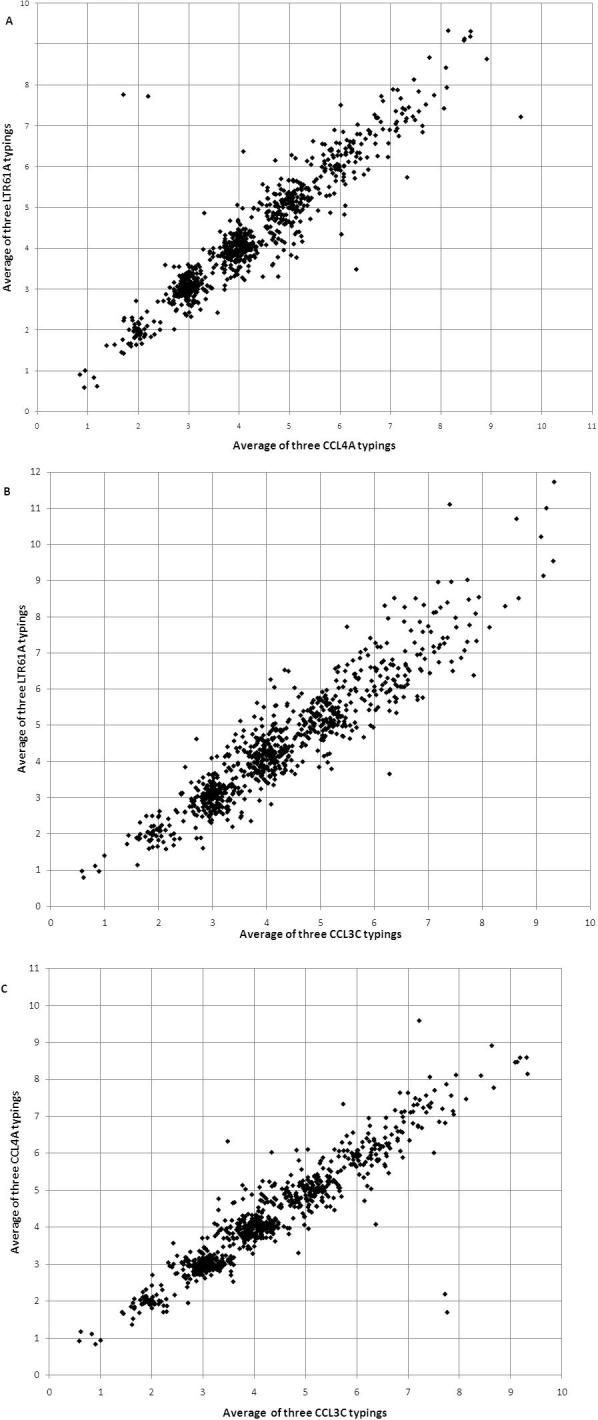
Concordance of mean unrounded copy number measures for 922 typed malaria samples for measurement using mean CCL4A with mean LTR61A (a), mean CCL3C with mean LTR61A (b) and mean CCL4A and CCL3C (c).

Copy number was measured from genomic DNA using the paralogue ratio test (PRT) previously described (Carpenter et al., 2011; Walker et al., 2009). Briefly, the PRT method is a PCR-based assay using a single pair of primers to simultaneously amplify two specific products in a single reaction, one from a single-copy reference locus and the other from the copy variable test locus of interest. The copy number of the test locus is then estimated from the ratio of test to reference PCR products. In European samples a single-tube triplex PRT assay has previously been described (Carpenter et al., 2011), using three independent PRT assays (CCL3C, CCL4A and LTR61A) to give three measures of copy number, which are then averaged into a single unrounded copy number value. In the African samples here the single tube triplex assay was performed in triplicate with different dyes; a FAM-labelled single tube triplex, a HEX-labelled triplex and a NED-labelled triplex, ultimately giving nine separate ratios of test to reference products. Two microsatellite PCRs were also performed for each sample as described previously (Walker et al., 2009). For each sample the products from the three triplex PCR reactions and the two microsatellites were mixed with 10 μl HiDi formamide with ROX-500 marker (Applied Biosystems, UK) for analysis. Fragment analysis was carried out by electrophoresis on an ABI3100 36 cm capillary using POP-4 polymer with an injection time of 30s at 2 kV (see Supplementary Fig. 1).

GeneMapper software (Applied Biosystems, UK) was used to extract the peak heights for the three systems and the ratio of test peak height to reference peak height was calculated for each sample for each dye independently. Copy number values were calculated by calibrating the ratios from each experiment with 5 Yoruba HapMap samples (http://www.coriell.org/) of established copy number [copy number (CN) = 2, NA19159; CN = 3, NA18870; CN = 4, NA19092; CN = 5, NA19171; and CN = 6, NA18503], which were included in every experiment in triplicate. Unrounded and calibrated copy numbers were compared and an average copy number value calculated. The average copy number data was then compared with microsatellite data to ascertain integer copy number (see further discussion in Supplementary results). In order to allow family based analysis, copy number haplotypes were determined in some families using segregation of microsatellite alleles.

### 2.4 Statistical analysis

Heritabilities of the parasite density, clinical episode and haemoglobin phenotypes were calculated by variance components analysis as implemented in SOLAR (Blangero and Almasy, 1997). The number of relative pairs contributing to each heritability analysis was calculated using the ‘relpairs’ command in SOLAR. The numbers of relative pairs were 1937 for parasite load, 2101 for clinical episodes and 1887 for haemoglobin. For the parasite load this included 669 parent–offspring comparisons, 847 sibling, or half sibling, 115 grandparent–grandchild, 62 avuncular or half avuncular and 16 first–cousin comparisons. For the clinical episodes this included 715 parent–offspring comparisons, 927 sibling or half sibling, 4 grandparent–grandchild, 73 avuncular or half avuncular and 16 first–cousin comparisons. For the haemoglobin this included 659 parent–offspring comparisons, 823 sibling or half sibling, 2 grandparent–grandchild, 48 avuncular or half avuncular and 16 first–cousin comparisons.

In order to evaluate the effect of total copy number on the malaria phenotypes within the family study the variance components analysis within SOLAR was used. This allows copy number to be modelled as a covariate in order to evaluate the proportion of variance attributable to it. Copy number was modelled as a covariate with copy number^2^, age, sex and age^2^ included, with all the interactions. Those terms that were non-significant were removed successively.

To allow family based analysis, parental information was collected and a total of 167 extended pedigrees compiled, as previously described (Carpenter et al., 2007, 2009). Associations between the clinical phenotypes and copy number haplotypes were tested by a quantitative transmission disequilibrium test (TDT) using the program QTDT version 2.1.3 (Abecasis et al., 2000). To control for any population stratification, we tested the within-family association in an orthogonal model including age and sex as covariates and environmental, polygenic heritability and additive major locus variance components. Total evidence of association was also modelled including age and sex as covariates and environmental, polygenic heritability and additive major locus variance components.

## 3 Results

### 3.1 Copy number measurements

**Fig. 1:**
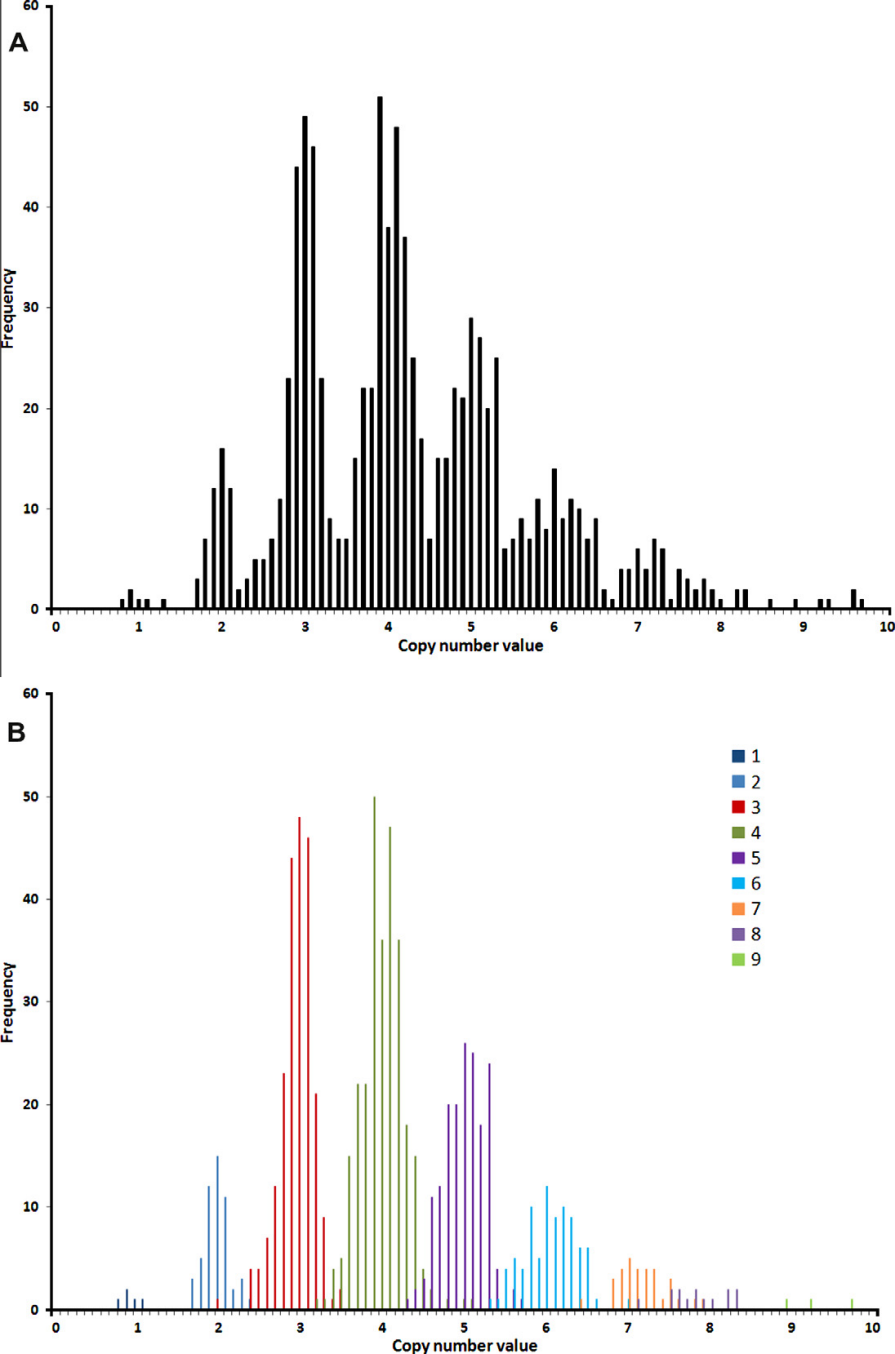
Distribution of average calibrated copy number values for the 922 typed malaria samples (a), and with microsatellite data also included (b) and represented by a different colour for each integer microsatellite.

In total there were 922 Tanzanian samples successfully genotyped for *CCL3L1* copy number. The distribution of unrounded copy numbers is shown in Fig. 1 (and as integers in Table 1) and suggests a range of 1–10 copies, with a mean copy number of about 4. In general the concordance between the nine PRT measurements is strong, with 76% of samples having all measurements concordant within 0.75 of the inferred integer, and the microsatellite data in agreement with the proposed integer from PRT in 89% of samples (see Fig. 1 and discussed in Supplementary results).

The overall standard deviation (normalised for copy number) of the full dataset was 0.07, which is small and consistent with previously typed European datasets (Carpenter et al., 2011; Walker et al., 2009), indicating the precision of the PRT measurements in estimating integer copy number. The mean and standard deviation, normalised standard deviations and predicted probability of error for the full dataset are shown in Supplementary Table 1. The data show that, at least for copy numbers less than 8, the means lie within 0.1 of the corresponding integer and that the standard deviations are sufficiently low that the probability of assigning a sample to the wrong integer class is also small. Whilst the estimates of the probability of error of copy number calling are small, there is an increase for higher copy numbers which is likely the result of the greater variance observed at the higher copies.

**Fig. 2:**
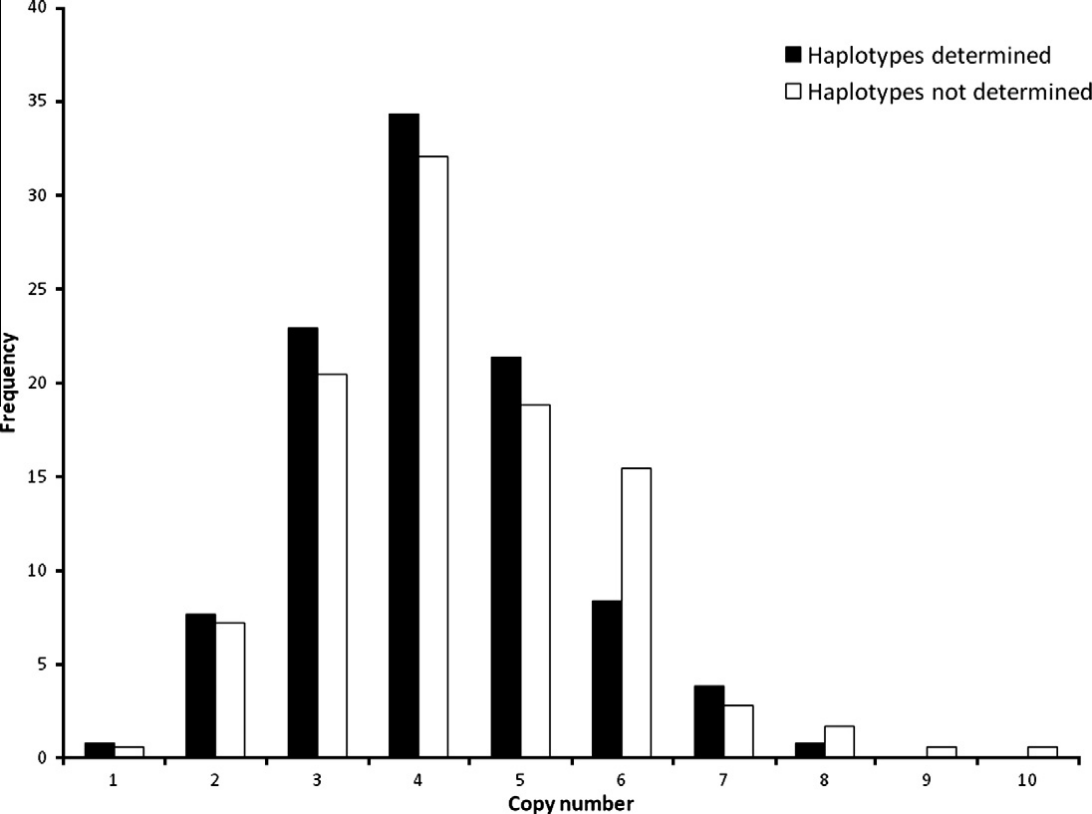
Distribution of total diploid copy numbers for subsets of samples for which haplotype composition could (black) or could not (white) be determined from segregation. The two distributions do not have significantly different means.

**Fig. 3:**
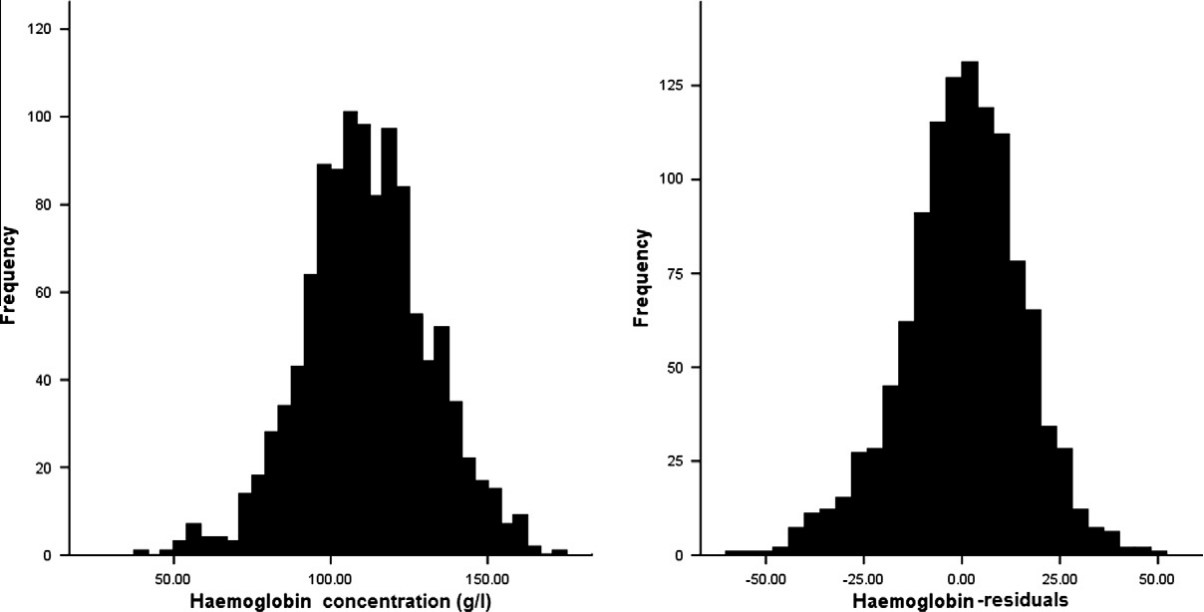
Distribution of the haemoglobin data before and after correction for age, sex and parasite load. The residuals generated from the correction were normally distributed and therefore no further transformation was made prior to genetic analysis.

The microsatellite alleles were used to allow determination of the copy number haplotypes by segregation. Unambiguous segregation was observed in 75/165 families (45%), which comprise a total of 591 informative individuals, comprised of 288 males, 303 females, an age range of between 1 and 86, and with a mean pedigree size of 14 individuals. Fig. 2 shows that the inference of haplotypes does not select a subset of individuals with distorted copy number distribution. In total there were 194 independent individuals that were phased for copy number, resulting in 388 phased copy number haplotypes. A breakdown of the more frequent copy number haplotypes, with the composite microsatellite alleles, is shown in Supplementary Table 2. The overall distribution of the haplotypes is shown in Table 2, demonstrating a range of 0–6 copies per chromosome and a mean of about 2.1 copies per chromosome. The data show that of the 388 haplotypes 179 are one of the 48 more frequent, leaving 209 (54%) haplotypes that are represented only once (see Supplementary Table 2). This suggests a high level of microsatellite allele diversity with a large number of different copy number haplotypes represented here.

### 3.2 Genetic analysis of association

The phenotypes used in this study were generated from longitudinal data collected in Nyamisati during 1993–1999. The epidemiology of the parasite load and the mean number of clinical episodes of malaria phenotypes have been discussed in detail previously (Carpenter et al., 2009), and for haemoglobin is shown in Table 3. The distribution of haemoglobin was significantly different (*p* < 0.001) between the sexes with a mean male haemoglobin concentration of 114.86 g/l ± 22.32, and a female concentration of 108.42 g/l ± 16.84. A significant positive relationship with age was also observed (*p* < 0.001), and therefore the haemoglobin data required age and sex correction, as well as correction for parasite load, prior to further genetic analysis.

As assessed by SOLAR the heritability (SE) of the haemoglobin data (g/l) was 0.302 (0.085) (*p* < 0.0001). The heritabilities of the parasite load phenotype (0.104 (SE = 0.052)) and of the mean number of clinical episodes of malaria phenotype (0.221(SE = 0.046)) have been measured previously (Carpenter et al., 2009). There is strong evidence from the QTDT analysis here that there is a polygenic component for all three malaria phenotypes: parasite load (*p* = 0.0066), mean number of clinical episodes of malaria (*p* = 2.5 × 10^−03^) and haemoglobin (*p* = 9.1 × 10^−04^).

The output of the variance components analysis within SOLAR for all three phenotypes are summarised and presented in Table 4. The variance components analysis found no evidence for a significant association of total copy number with any of the malaria phenotypes. However there was some evidence for an association of copy number squared and haemoglobin (*p* = 0.035), which suggests that any effect of total copy number on haemoglobin levels is non-linear, and also a weak correlation with age * sex (*p* = 0.049) was observed. Weak evidence for an effect of copy number squared was also observed with mean number of clinical episodes of malaria (*p* = 0.041), again suggesting that any effect of total copy number on mean number of clinical malaria episodes is non-linear. There was also evidence with the number of clinical malaria episodes phenotype for a strong effect of age (*p* = 0.001) and the interaction sex * total copy number (*p* = 0.034).

Quantitative TDT analysis was performed using haplotypes that were generated through segregation using the microsatellite alleles that are situated within the copy variable region, but this is not possible for all families, and therefore the analysis is on a restricted sample. A summary of the output of these analyses are presented in Table 5, and show that whilst there were some nominally significant observations with copy number haplotypes, only a total model of association with haemoglobin has a borderline significance with the global test, and therefore is the only one for which haplotype data should be subsequently examined. More specifically, a positive association with haemoglobin was observed with the 2-copy haplotype (*p* = 0.021). There was also weak evidence for a negative association with the 1-copy haplotype (*p* = 0.045) but neither of these observations were supported when including family structure and would not be significant after multiple testing.

## 4 Discussion

The PRT system for copy number measurement of *CCL3L1* has previously been shown to have a high degree of accuracy in integer copy number prediction (Carpenter et al., 2011; Walker et al., 2009). Here we adapted the PRT methodology for use with African samples. African samples have previously been measured for *CCL3L1* copy number using another measurement system (Gonzalez et al., 2005), and predicted to have a range of 2–14. Our data suggests that the range in Tanzania is 1–10; however, the integer copy numbers in this study were assigned from PRT data which has sufficient accuracy to identify discrete clusters consistent with microsatellite data, whereas the previous data did not fall into discrete clusters for integer determination (Gonzalez et al., 2005) [their Supplementary Fig. S13], making it difficult to compare copy number ranges.

This study is the first to examine association of susceptibility to malaria phenotypes with *CCL3L1* copy number. We used a well-documented population from Tanzania and a family based study to investigate the copy number association. A family-based approach overcomes problems of population admixture and stratification that can influence case-control studies. The malaria phenotypes result from data collected over a number of years, the use of multiple time-points should provide more information than a single time point and thus a better portrayal of an individual’s susceptibility.

The heritability of haemoglobin level was 0.316, which is a good value for genetic analysis. Whilst we have not been able to incorporate household effects into the heritability estimate due to the SOLAR method, the value is generated from longitudinal data, which generates a more robust measurement of heritability than a single time-point. This value is similar to previous studies for haemoglobin heritiabilities (Garner et al., 2000; Sala et al., 2008), and suggests a high genetic contribution to haemoglobin levels. The heritabilities of parasite load and mean number of clinical episodes of malaria data have been previously reported and are lower than that for haemoglobin (Carpenter et al., 2009), suggesting that the sample size required to detect genes that contribute to parasite levels and clinical episodes will be higher than for haemoglobin (Carpenter et al., 2009).

We found limited evidence for an influence of *CCL3L1* copy number on malaria phenotypes. The most interesting observation was with haemoglobin levels, where there was evidence that total copy number was shown to have a weak, non-linear association, and the allele specific analysis identified a positive association with a two copy haplotype. Previous studies have found that high levels of MIP-1α inhibit haematopoiesis, and therefore it is possible that at lower copy numbers there is a correspondingly reduced level of MIP-1α, a significantly reduced inhibition of haematopoiesis, and ultimately less influence on malarial anaemia.

The evidence for the association between total copy number and haemoglobin levels is weak, and is not significant after correction for multiple testing. This may in part be due to lack of power, especially with the QTDT analysis where the sample size was significantly reduced. However, the evidence for a polygenic component to haemoglobin levels is strong, and it is likely therefore that some of the genetic factors influencing this quantitative phenotype will have small, but significant, biological effects, as has been observed previously for malarial anaemia (Anyona et al., 2011; Awandare et al., 2009; Ong’echa et al., 2011 and reviewed in Perkins et al. (2011)) and observed in other complex traits (Altmüller et al., 2001; Maher, 2008). The evidence for association is similar for the haemoglobin data both with and without the correction for parasite load, as is described in Section 2. This correction applies to relatively few individuals in our data-set, and to ascertain the role of malaria on the haemoglobin phenotype would require more detailed investigation. This population has not been screened for other genetic determinants that may influence haemoglobin status. Furthermore, there are a number of causes of anaemia in malaria endemic areas, and whilst persistent malaria infection is a major factor there are other important contributory causes including co-infections with HIV-1 (Davenport et al., 2010), and malnutrition.

Cerebral malaria results from the sequestration of parasitised red blood cells and leucocytes in the brain. CCR5, the receptor for MIP-1α is required for trafficking of leucocytes and CD8+ cells to the brain and thus for cerebral malaria to develop in infected mice, and it has been observed that CCR5 deficient mice are resistant to cerebral malaria (Belnoue et al., 2003). An upregulation of CCR5 expression and also an upregulation of MIP-1α in the brains and lungs of malaria infected mice has been observed (Belnoue et al., 2008). This study population does not have any cases of cerebral malaria, but it would certainly be of interest to investigate *CCL3L1* copy number in a study population that presented with cerebral malaria as a phenotype.

Furthermore malaria infection during pregnancy is associated with poor birth outcomes, due, in part, to sequestered parasites in the placenta and results in monocyte penetration into the placental intervillous space (Ordi et al., 1998) and a localised immune response (Moormann et al., 1999). Malaria infected mothers have been shown to have a 3-fold increased expression of CCR5 (Tkachuk et al., 2001), and MIP-1α has been shown to be associated with placental malaria and with monocyte density (Abrams et al., 2003). Therefore further investigations of *CCL3L1* copy number with placental malaria may also be of interest.

The range of *CCL3L1* copy numbers observed in this African population is greater than that observed with European populations, though it is not fully understood how this divergence has evolved or been maintained. It is probable that the observed diversity of copy number haplotypes has been generated both by the expansion and/or reduction of copy variable repeats units combined with the mutation of microsatellites within each repeat unit. Mice do not have an orthologue of *CCL3L1* and have only a *CCL3* orthologue. Chimpanzees have been shown not to be copy variable for *CCL3L1* (Perry et al., 2008) (and our unpublished data). It is thus possible that this locus was at high copy number in the human ancestral population and has reduced to low average copy number after a bottleneck associated with European divergence, or there is selection acting, either upon African populations to maintain higher copy numbers or in Europeans for a lower copy number. Our data shows that the African population sampled here does show a large range of total *CCL3L1* copy numbers and that the constituent alleles are variable and diverse. This is the first time that African *CCL3L1* copy number haplotypes have been described, and are in contrast to our observation of European *CCL3L1* copy number haplotypes described previously (Walker et al., 2009), and also for the 30 HapMap CEPH trios which show significantly less variability of microsatellite alleles in phased haplotypes (see Supplementary Table 3). This is not unexpected, as African populations have greater diversity than European populations at a number of loci (The 1000 Genomes Project Consortium, 2010). However, whether the contrast of population specific *CCL3L1* haplotype diversity is a consequence of a founder effect or selection cannot be simply inferred from our data.

To conclude, we have found weak evidence for an association with lower *CCL3L1* copy numbers and protection from anaemia. The data also demonstrate that African populations maintain a wide copy number distribution of *CCL3L1* on diverse haplotype backgrounds. It is still unclear whether this observed difference in *CCL3L1* copy number diversity is generated by selection.

## Figures and Tables

**Table 1:** Distribution of integer copy numbers.

Copy number	Number
0	0
1	6
2	55
3	227
4	284
5	191
6	100
7	36
8	17
9	5
10	1

Total	922

**Table 2:** Distribution of copy number haplotypes.

Copy number haplotype	Number
0	1
1	108
2	174
3	72
4	22
5	9
6	2

Total	388

**Table 3:** Number of individuals with recorded haemoglobin levels (g/l) in Nyamisati by year.

Year	*n*^a^	Range (g/l) (all individuals)	Geometric mean (95% CI)
1994	292	53–163	104.05 (98.49–109.6)
1995	652	16–171	107.69 (101.78–113.59)
1996	13^b^	43–126	88.53 (83.35–93.71)
1997	0		
1998	152	48–153	112.09 (109.34–114.83)
1999	847	49–176	112.31 (107.08–117.53)

a *n* = number of individuals sampled.

b In 1996 only haemoglobin levels were tested only on clinical suspicion (pregnancy and/or clinical signs of anaemia).

**Table 4:** Variance components analysis using SOLAR. Values presented are *p* values for the different components included. Bold figures correspond to p < 0.05.

Covariates	Parasite load	Clinical episodes	Haemoglobin
Age	n/s^a^	**0.001**	n/s
Sex	n/s	n/s	n/s
Total copy number	n/s	0.098	n/s
Age^b^	n/s-removed^b^	**0.001**	n/s-removed
Sex^b^	n/s-removed	n/s-removed	n/s-removed
Total copy number^b^	n/s-removed	**0.041**	**0.035**
Age * sex	n/s-removed	0.059	**0.049**
Sex * total copy number	n/s-removed	**0.034**	n/s-removed
Sex * total copy number^b^	n/s-removed	0.063	n/s-removed

a Not significant.

b Not significant, and removed from the final analysis.

**Table 5:** Tests of association between copy number haplotypes and the quantitative malaria phenotypes. Values presented are *p* values for total and within-family tests of association using QTDT. Significant *p* values are in bold.

	Parasite load		Clinical episodes		Haemoglobin	
	Total association	Within-family association	Total association	Within-family association	Total association	Within-family association
Global score	n/s	n/s	n/s	n/s	0.063	n/s

*Copy number haplotype*						
0	**0.0237**	n/s	n/s	n/s	n/s	n/s
1	n/s	n/s	**0.026**	n/s	**0.045**	n/s
2	n/s	n/s	n/s	n/s	**0.021**	n/s
3	n/s	n/s	n/s	n/s	n/s	n/s
4	n/s	n/s	n/s	**0.028**	n/s	n/s
5	n/s	n/s	**0.029**	n/s	n/s	n/s
6	n/s	n/s	n/s	n/s	n/s	n/s
7	n/s	n/s	n/s	n/s	n/s	n/s
