# Supplementary material for: CCL3L1 copy number and susceptibility to malaria
Source: Infect Genet Evol. 2012 Jul;12(5):1147–54. doi: 10.1016/j.meegid.2012.03.021 (PMC3401375; doi:10.1016/j.meegid.2012.03.021)
Supplement: Supplementary data [file mmc1.doc]

**SUPPLEMENTARY INFORMATION FOR;**

***CCL3L1* copy number and susceptibility to malaria**

Danielle Carpenter^1*^, Ingergerd Rooth^2^, Anna Färnett^3^, John AL Armour^1^, Marie-Anne Shaw^4^

^1^Centre for Genetics and Genomics and School of Biology, University of Nottingham, Nottingham, NG7 2UH, UK

^2^Nyamisati Malaria Research, Rufiji, National Institute for Medical Research, Dar-es-Salaam, Tanzania

^3^Infectious Disease Unit, Department of Medicine Solna, Karolinska Institutet, Stockholm, Sweden

^4^Institute of Integrative and Comparative Biology, Faculty of Biological Sciences, University of Leeds, Leeds LS2 9JT, UK

^*^Corresponding author D. Carpenter PhD; Tel.: +44 115 823 0309; fax: +44 115 8230338

E-mail address: [danielle.carpenter@nottingham.ac.uk](mailto:danielle.carpenter@nottingham.ac.uk)

This is the first time that African samples have been genotyped for *CCL3L1*/*CCL4L1* copy number using the PRT method. Consequently the distribution of unrounded copy number, probability of error and accuracy of the copy number genotyping are discussed here in detail.

**Supplementary results**

The paralogue ratio test (PRT) was used to genotype the copy number of the *CCL3L1*/*CCL4L1* copy variable region in a total of 1,050 samples of African origin of which 121 appear to contain no DNA.

For each sample three independent triplex PRT assays were performed and the products used to ascertain integer copy number of *CCL3L1*. The PRT assays therefore generate a total of nine separate ratios of test peak height to reference peak height. The two microsatellite assays were combined with the three PRT reactions into a single capillary for electrophoresis of each sample to complement the PRT copy number measurement and improve precision without an increase in the cost. Mean unrounded calibrated copy numbers were generated for each PRT system (CCL3C, CCL4A and LTR61A), as well as a single overall mean value. These values were then compared within a sample to generate an inferred unrounded copy number value, which was then compared to the integer copy number predicted from the microsatellite data.

In general the three independent PRT assays assigned concordant measurements of copy number; all none unrounded copy numbers were within 0.5 of the inferred integer value for 56% of samples (523/929), and within 0.75 of the inferred integer for 76% of samples (706/929). The agreement between the PRT systems is shown in supplementary figure S1. As germ-line copy numbers are integers, accuracy of the measurement system can be inferred from clustering of data around the underlying integer values. The distribution of the raw data is shown in figure 1a and clearly shows PRT values clustering around the inferred integers. Furthermore, comparison with the predicted integer copy number value generated from microsatellite data adds support for the predicted integer copy number values from PRT (see figure 1b). In the majority of cases (89%) the integer copy number value predicted from the microsatellite data agreed with that from the PRT data (828/929). There were 69 samples where the integer copy number value predicted from the microsatellite data differed from that generated from the mean PRT data by 1 copy number; in 57 of these cases the integer predicted from the microsatellite data was in agreement with at least seven of the nine separate PRT measurements, and the disagreement with the mean was due to one or two outlier PRT measurements. There were 27 samples in which there were no informative microsatellite data to complement the PRT measurements. In these cases only, the copy number was determined from the PRT value only. There were seven cases where the PRT measurements and the microsatellites were in such conflict that no integer copy number could not inferred, and these samples are omitted from further analysis, leaving a total of 922 samples genotyped for *CCL3L1*/*CCL4L1* copy number.
