# Supplementary material for: CCL3L1 copy number and susceptibility to malaria
Source: Infect Genet Evol. 2012 Jul;12(5):1147–54. doi: 10.1016/j.meegid.2012.03.021 (PMC3401375; doi:10.1016/j.meegid.2012.03.021)
Supplement: Supplementary Table 1 [file mmc2.doc]

| Supplementary Table S1; Mean, deviations and error rates for average calibrated copy number values | | | | | | |
| --- | --- | --- | --- | --- | --- | --- |
| Copy Number | No | Mean | Standard deviation | Normalised Standard deviation | Specific deviation | Predicted error rate |
| 0 | 0 |  |  |  |  |  |
| 1 | 6 | 1.013 | 0.189 | 0.189 | 0.189 | 8.2x10^-03^ |
| 2 | 55 | 1.987 | 0.147 | 0.074 | 0.106 | 7.3x10^-04^ |
| 3 | 227 | 2.972 | 0.214 | 0.069 | 0.119 | 2x10^-02^ |
| 4 | 282 | 4.004 | 0.259 | 0.065 | 0.129 | 5.4x10^-02^ |
| 5 | 188 | 4.992 | 0.292 | 0.058 | 0.131 | 8.7x10^-02^ |
| 6 | 100 | 5.992 | 0.374 | 0.062 | 0.152 | 0.182 |
| 7 | 36 | 7.116 | 0.331 | 0.047 | 0.132 | 0.154 |
| 8 | 17 | 7.842 | 0.401 | 0.050 | 0.153 | 0.246 |
| 9 | 5 | 9.327 | 0.330 | 0.037 | 0.164 | 0.306 |
| 10 | 1 |  |  |  |  |  |
