# Supplementary material for: CCL3L1 copy number and susceptibility to malaria
Source: Infect Genet Evol. 2012 Jul;12(5):1147–54. doi: 10.1016/j.meegid.2012.03.021 (PMC3401375; doi:10.1016/j.meegid.2012.03.021)
Supplement: Supplementary Table 2 [file mmc3.doc]

| Supplementary Table S2. Distribution of the most common (n=48) microsatellite haplotypes in the African population defined by the composite microsatelite alleles | | | | | | | | | |
| --- | --- | --- | --- | --- | --- | --- | --- | --- | --- |
| CN^a^ | TTAT alleles^b^ | | | | TATC alleles^c^ | | | | Number |
| 1 | 203 |  |  |  | 398 |  |  |  | 3 |
| 1 | 203 |  |  |  | 400 |  |  |  | 5 |
| 1 | 203 |  |  |  | 405 |  |  |  | 14 |
| 1 | 203 |  |  |  | 410 |  |  |  | 2 |
| 1 | 225 |  |  |  | 400 |  |  |  | 8 |
| 1 | 225 |  |  |  | 405 |  |  |  | 3 |
| 1 | 225 |  |  |  | 412 |  |  |  | 5 |
| 1 | 225 |  |  |  | 414 |  |  |  | 2 |
| 1 | 227 |  |  |  | 405 |  |  |  | 3 |
| 1 | 229 |  |  |  | 396 |  |  |  | 2 |
| 1 | 229 |  |  |  | 398 |  |  |  | 5 |
| 1 | 229 |  |  |  | 405 |  |  |  | 4 |
| 1 | 229 |  |  |  | 410 |  |  |  | 2 |
| 1 | 233 |  |  |  | 405 |  |  |  | 4 |
| 1 | 233 |  |  |  | 412 |  |  |  | 7 |
| 2 | 203 | 203 |  |  | 398 | 400 |  |  | 2 |
| 2 | 203 | 203 |  |  | 398 | 405 |  |  | 4 |
| 2 | 203 | 203 |  |  | 400 | 400 |  |  | 4 |
| 2 | 203 | 203 |  |  | 400 | 405 |  |  | 6 |
| 2 | 203 | 203 |  |  | 405 | 405 |  |  | 4 |
| 2 | 203 | 225 |  |  | 400 | 405 |  |  | 2 |
| 2 | 203 | 225 |  |  | 405 | 405 |  |  | 2 |
| 2 | 203 | 229 |  |  | 400 | 400 |  |  | 2 |
| 2 | 203 | 229 |  |  | 400 | 405 |  |  | 4 |
| 2 | 203 | 229 |  |  | 405 | 405 |  |  | 4 |
| 2 | 205 | 229 |  |  | 384 | 405 |  |  | 4 |
| 2 | 223 | 227 |  |  | 405 | 410 |  |  | 2 |
| 2 | 225 | 225 |  |  | 384 | 405 |  |  | 3 |
| 2 | 225 | 225 |  |  | 396 | 408 |  |  | 5 |
| 2 | 225 | 225 |  |  | 396 | 410 |  |  | 3 |
| 2 | 225 | 225 |  |  | 396 | 412 |  |  | 2 |
| 2 | 225 | 225 |  |  | 398 | 408 |  |  | 3 |
| 2 | 225 | 225 |  |  | 398 | 410 |  |  | 5 |
| 2 | 225 | 225 |  |  | 405 | 405 |  |  | 2 |
| 2 | 225 | 229 |  |  | 405 | 405 |  |  | 5 |
| 2 | 225 | 229 |  |  | 405 | 410 |  |  | 5 |
| 2 | 225 | 229 |  |  | 412 | 414 |  |  | 2 |
| 2 | 225 | 233 |  |  | 405 | 405 |  |  | 8 |
| 2 | 225 | 233 |  |  | 412 | 414 |  |  | 2 |
| 2 | 229 | 229 |  |  | 392 | 405 |  |  | 4 |
| 2 | 229 | 229 |  |  | 400 | 408 |  |  | 2 |
| 2 | 231 | 233 |  |  | 410 | 416 |  |  | 2 |
| 3 | 203 | 203 | 225 |  | 405 | 405 | 405 |  | 3 |
| 3 | 203 | 225 | 225 |  | 400 | 405 | 405 |  | 3 |
| 3 | 225 | 225 | 225 |  | 405 | 405 | 410 |  | 2 |
| 3 | 225 | 225 | 231 |  | 405 | 410 | 414 |  | 2 |
| 3 | 225 | 225 | 233 |  | 398 | 405 | 405 |  | 5 |
| 4 | 203 | 225 | 225 | 229 | 398 | 400 | 405 | 405 | 2 |
| ^a^Haplotype copy number  ^b^Observed alleles for TTAT microsatellite are 203bp, 205bp, 225bp, 227bp, 229bp, 231bp, 233bp. ^c^Observed alleles for TATC microsatellite are 384bp, 394bp, 396bp, 398bp, 400bp, 402bp, 404bp, 406bp, 408bp, 410bp, 412bp, 414bp, 416bp, 418bp, 420bp | | | | | | | | | |
